# Algorithm to quantify nuclear features and confidence intervals for classification of oral neoplasia from high-resolution optical images

**DOI:** 10.1117/1.JMI.7.5.054502

**Published:** 2020-09-21

**Authors:** Eric C. Yang, David R. Brenes, Imran S. Vohra, Richard A. Schwarz, Michelle D. Williams, Nadarajah Vigneswaran, Ann M. Gillenwater, Rebecca R. Richards-Kortum

**Affiliations:** a Baylor College of Medicine, Houston, Texas, United States; b Rice University, Department of Bioengineering, Houston, Texas, United States; c The University of Texas, MD Anderson Cancer Center, Department of Pathology, Houston, Texas, United States; d The University of Texas, School of Dentistry at Houston, Department of Diagnostic and Biomedical Sciences, Houston, Texas, United States; e The University of Texas, MD Anderson Cancer Center, Department of Head and Neck Surgery, Houston, Texas, United States

**Keywords:** semantic segmentation, parameter estimation, microscopy, oral cancer

## Abstract

**Purpose:** *In vivo* optical imaging technologies like high-resolution microendoscopy (HRME) can image nuclei of the oral epithelium. In principle, automated algorithms can then calculate nuclear features to distinguish neoplastic from benign tissue. However, images frequently contain regions without visible nuclei, due to biological and technical factors, decreasing the data available to and accuracy of image analysis algorithms.

**Approach:** We developed the nuclear density-confidence interval (ND-CI) algorithm to determine if an HRME image contains sufficient nuclei for classification, or if a better image is required. The algorithm uses a convolutional neural network to exclude image regions without visible nuclei. Then the remaining regions are used to estimate a confidence interval (CI) for the number of abnormal nuclei $\mathrm{per}\;{\mathrm{mm}}^{2}$, a feature used by a previously developed algorithm (called the ND algorithm), to classify images as benign or neoplastic. The range of the CI determines whether the ND-CI algorithm can classify an image with confidence, and if so, the predicted category. The ND and ND-CI algorithm were compared by calculating their positive predictive value (PPV) and negative predictive value (NPV) on 82 oral biopsies with histopathologically confirmed diagnoses.

**Results:** After excluding the images that could not be classified with confidence, the ND-CI algorithm had higher PPV (65% versus 59%) and NPV (78% versus 75%) than the ND algorithm.

**Conclusions:** The ND-CI algorithm could improve the real-time classification of HRME images of the oral epithelium by informing the user if an improved image is required for diagnosis.

## 1 Introduction

In recent years, *in vivo* optical microscopy devices that visualize subcellular tissue features have been utilized to distinguish cancerous and precancerous tissue from benign tissue at a diverse range of anatomic sites.^1^^–^^4^ Compared to traditional tissue biopsies and histopathologic analysis, these devices are noninvasive and provide real-time results. Their potential application spans the spectrum of cancer care, from the diagnosis and surveillance of premalignant lesions, to margin delineation during surgical resections, and monitoring patients following treatment for recurrence.

Reflectance confocal microscopy, confocal laser microendoscopy, and high-resolution microendoscopy (HRME) are examples of optical microscopy technologies that have been shown to visualize the morphology of the nuclei in the oral epithelium.^5^^–^^8^ Because abnormal nuclear morphology is a key hallmark of oral neoplasia, numerous clinical studies have demonstrated their potential to diagnose oral neoplasia.^9^^–^^16^

To reduce subjectivity in image interpretation, automated algorithms have been developed to calculate statistical features of nuclei that correlate with neoplasia, like nuclear density, mean nuclear size and variance, nuclear eccentricity, and the nuclear-to-cytoplasm ratio.^17^^–^^19^ These features can then be used as inputs to machine learning algorithms that distinguish neoplastic tissue from benign tissue. For example, studies have shown that a feature called the number of abnormal nuclei $\mathrm{per}\;{\mathrm{mm}}^{2}$, calculated from HRME images, can distinguish oral cancer and high-grade dysplasia from benign tissue with high accuracy (the ND algorithm).^19^^,^^20^

One limitation of *in vivo* microscopy in the oral mucosa is that images frequently contain regions without reliable information about nuclei. For example, leukoplakias, which are the most common oral premalignant lesion, contain a highly scattering keratin layer that overlies the epithelium and can prevent visualization of the subsurface nuclei with good contrast.^5^^,^^6^^,^^8^ Leukoplakias may also contain nonkeratinized regions that are more amenable to analysis.^21^ Regions without visible nuclei can also occur due to technical imaging challenges. It can be difficult to maintain steady contact between confocal imaging probes, which are often rigid and bulky, and the oral mucosa, particularly in deeper areas of the mouth and in clinical settings where patients are not sedated.^5^^,^^6^ This can lead to motion blur or poor focus in all or a portion of an image. HRME, which utilizes a thin, flexible fiber-optic probe, is less prone but not immune to these issues. Other causes of poor image contrast include uneven tissue surfaces, which can result in saturation of the image sensor, low signal intensity, and air bubbles in saliva.

Due to the variety of possible causes, image analysis algorithms like the ND algorithm do not effectively exclude regions without visible nuclei, leading to feature values that do not accurately represent the tissue. Moreover, even if such regions could be excluded, analyzing an image with only a small region of reliable information may not yield sufficient information to make a diagnostic prediction with high confidence. It is not clear how to quantify the impact of the uncertainty in feature extraction on prediction accuracy.

In this paper, we develop the nuclear density-confidence interval (ND-CI) algorithm to classify high-resolution images of the oral epithelium, including those containing regions without visible nuclei, as neoplastic or benign. The algorithm extracts information only from regions in which nuclei are adequately visualized, and makes diagnostic predictions only if accurate prediction is likely. The diagnostic performance of the ND-CI algorithm is then compared to the ND algorithm, using histopathology results from 82 biopsies acquired from patients with oral lesions, as the gold standard.

## 2 Methods

### 2.1 High-Resolution Microendoscopy

**Fig. 1:**
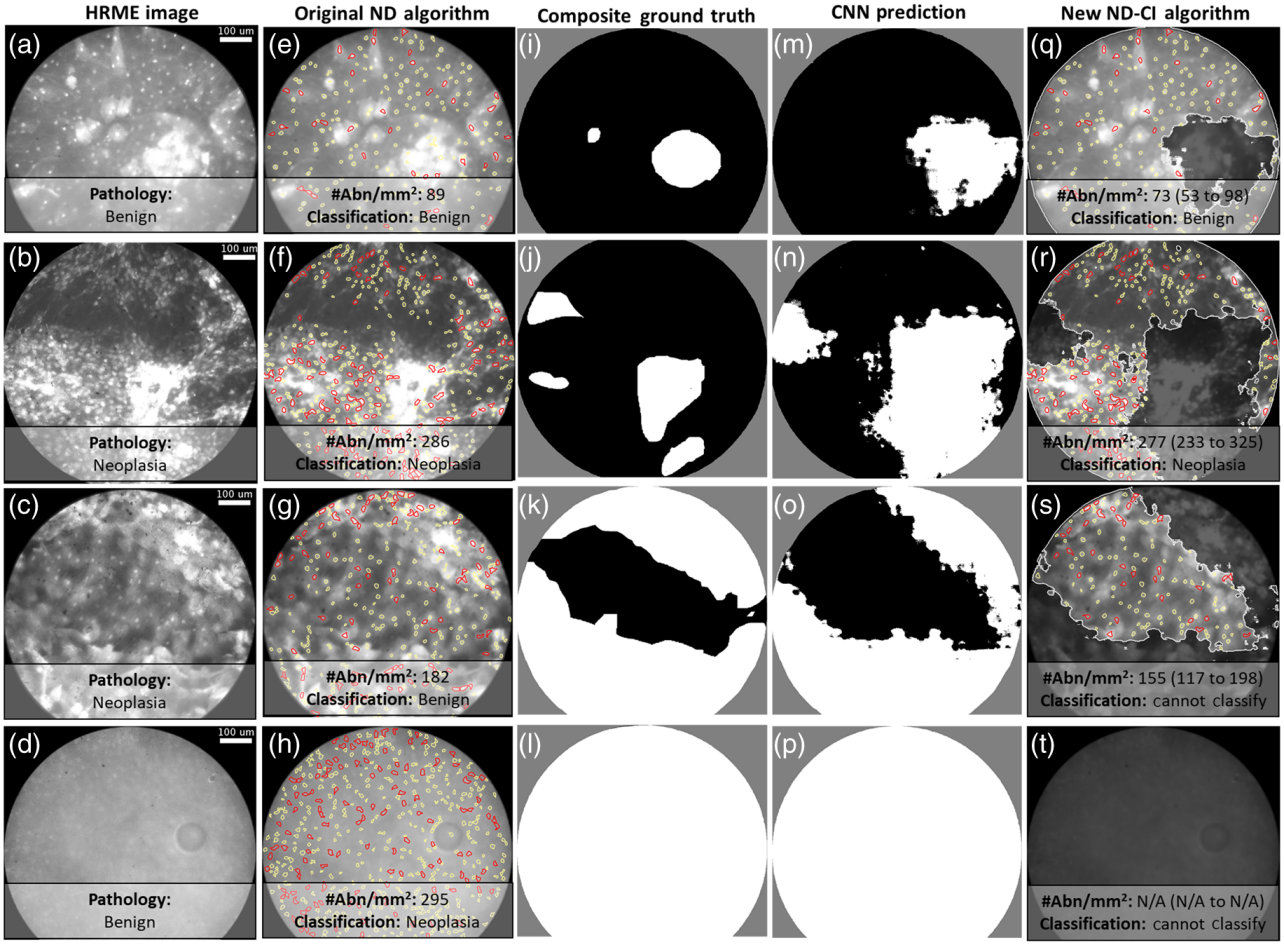
Comparison of the ND and ND-CI algorithms on example HRME images from the test set. (a)–(d) Representative HRME images and corresponding histopathological diagnoses. (e)–(h) Results of the original ND algorithm, which does not exclude regions without visible nuclei. Yellow outlines represent nuclei classified as normal, and red outlines represent nuclei classified as abnormal. The number of abnormal nuclei $\mathrm{per}\;{\mathrm{mm}}^{2}$ ($\#\mathrm{Abn}/{\mathrm{mm}}^{2}$) and classification result are also shown. (i)–(l) Ground truth masks based on manual segmentation by three raters, delineating regions with visible nuclei (black) and without visible nuclei (white). Background areas are gray. (m)–(p) The predictions of the CNN, using the same color scheme as (i)–(l). (q)–(t) Results of the new ND-CI algorithm, which uses the CNN predictions [(m)–(p)] to exclude regions without visible nuclei (dimmed for ease of visualization). The color scheme is the same as (e)–(h). The $\#\mathrm{Abn}/{\mathrm{mm}}^{2}$ in the visible nuclei regions, the 90% CIs for the $\#\mathrm{Abn}/{\mathrm{mm}}^{2}$, and classification results are also shown.

The HRME uses a fluorescence microscope and a 790-μm diameter fiber-optic probe to acquire high-resolution images of the nuclei in the superficial oral epithelium.^19^ The raw images are 1280×960  pixels with a lateral resolution of 4.4  μm^19^ and are automatically cropped to the active area of the fiber-optic probe, typically about ∼960×960  pixels. Neoplasia, which is diagnosed histopathologically, is associated with crowded, large, eccentric, and pleomorphic nuclei.^22^ Figure 1 shows four examples of HRME images acquired from oral lesions. The circular fields of view (FOVs) correspond to the areas imaged with the fiber-optic probe; superficial epithelial nuclei are visible as white round/oval structures. The image shown in Fig. 1(a) was acquired from tissue that was histopathologically diagnosed as benign; the nuclei are small, round, and evenly spaced. In contrast, the image shown in Fig. 1(b) was acquired from histopathologically confirmed neoplasia; the nuclei are crowded, large, eccentric, and more variable in size and shape.

### 2.2 ND Algorithm

The ND algorithm calculates a feature called the number of abnormal nuclei $\mathrm{per}\;{\mathrm{mm}}^{2}$ from HRME images.^19^ The algorithm identifies individual nuclei within an image and classifies each as normal or abnormal based on its size and eccentricity; the number of abnormal nuclei $\mathrm{per}\;{\mathrm{mm}}^{2}$ is simply the density of these abnormal nuclei. In a previous study, sites in patients with oral lesions were imaged with the HRME, and the number of abnormal nuclei $\mathrm{per}\;{\mathrm{mm}}^{2}$ was used to distinguish neoplastic from non-neoplastic sites.^13^ Since that study, an updated version of the HRME instrumentation was developed.^19^ We conducted a similar study (unpublished) using the updated instrumentation to update the classification thresholds; in this second study, a decision boundary of 189 abnormal nuclei $\mathrm{per}\;{\mathrm{mm}}^{2}$ was found to optimally distinguish neoplastic from benign tissue.

The results of the ND algorithm on the four examples are shown in the second column of Fig. 1(e)–1(h); nuclei classified as normal are outlined in yellow, and nuclei classified as abnormal are outlined in red. The number of abnormal nuclei $\mathrm{per}\;{\mathrm{mm}}^{2}$ in the first two images was 89 and 286 [Figs. 1(e) and 1(f)], respectively, indicating that the algorithm result agreed with the histopathological diagnosis in both cases.

Unfortunately, HRME images often contain regions without visible nuclei, as illustrated in the third column of Figs. 1(i)–1(l)]. White represents regions without visible nuclei, black represents regions where nuclei are clearly visible, and gray represents background outside the active area of the probe. The first image has two regions where the image intensity exceeded the camera dynamic range and the image is saturated; these regions likely correspond to areas of tissue with overlying debris that is highly scattering [Fig. 1(i)]. The second image has a saturated region at the bottom right and a dim region, possibly due to poor contact between the probe and tissue surfaces, near 10 o’clock [Fig. 1(j)]. The third image [Fig. 1(c)] was obtained from a hyperkeratinized region, with a horizontal band of nuclei visible through the center [Fig. 1(k)]. The final image [Fig. 1(d)] was obtained from tissue with visible nuclei; however, the entire image had very poor contrast, possibly due to a low HRME LED battery, and it is difficult to visualize the morphology of individual nuclei.

The ND algorithm attempted to segment all regions of the images, including dim, saturated, and keratinized regions; information from these regions resulted in misclassification of the images shown in Figs. 1(c) and 1(d). The image in Fig. 1(c) was histopathologically diagnosed as neoplasia but was misclassified as benign [Fig. 1(g)]. The image in Fig. 1(d) was histopathologically benign but was misclassified as neoplastic [Fig. 1(h)], because many abnormal nuclei were erroneously identified.

### 2.3 ND-CI Algorithm

**Fig. 2:**
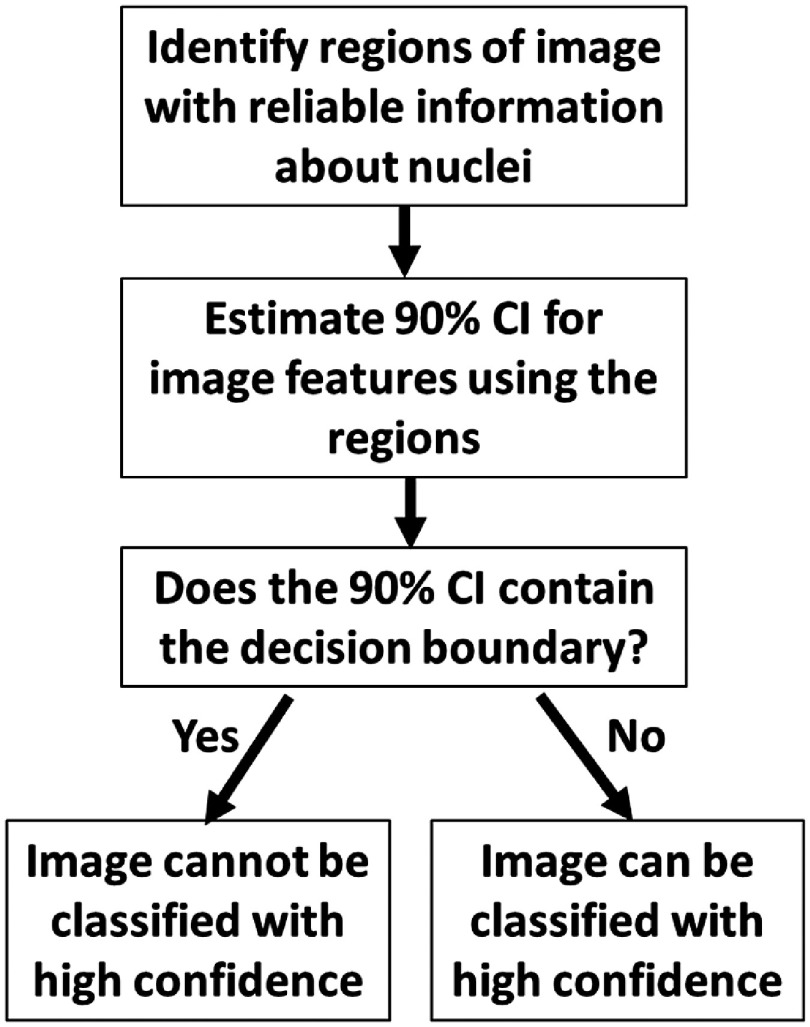
Flowchart of the ND-CI algorithm.

The ND-CI algorithm (Fig. 2) addresses these limitations of the ND algorithm. The first step is to identify the image regions containing visible nuclei. Probabilistic methods are then used to calculate a 90% confidence interval (CI) for the true value of the number of abnormal nuclei $\mathrm{per}\;{\mathrm{mm}}^{2}$ using only these regions. The CI is then compared to the decision boundary of 189: if the full range is above the decision boundary $\left({\mathrm{CI}}_{\text{high}}>{\mathrm{CI}}_{\text{low}}>189\right)$, then the image is classified as neoplastic; if the full range is below the decision boundary $\left({\mathrm{CI}}_{\text{low}}<{\mathrm{CI}}_{\text{high}}<189\right)$, then the image is classified as benign. However, if the CI includes the decision boundary $\left({\mathrm{CI}}_{\text{low}}<189<{\mathrm{CI}}_{\text{high}}\right)$, then the image cannot be classified with confidence because the CI spans more than one diagnostic category.

#### 2.3.1 HRME image acquisition

To develop the ND-CI algorithm, a databank of 811 HRME images was acquired from the oral mucosa of 169 patients with oral lesions as part of protocols approved by the Institutional Review Boards at Rice University, MD Anderson Cancer Center, and the UTHealth School of Dentistry, all in Houston, Texas. Written informed consent was acquired before imaging. A head and neck surgeon (A. G.) or an oral pathologist (N. V.) examined the patients, who were presenting for regularly scheduled clinic visits or for surgical resection of their oral lesion(s), then saved HRME images at sites of interest. After imaging, clinically indicated biopsies or surgical resections were performed, and the resulting tissue specimens were processed and diagnosed histopathologically in accord with standard clinical procedures and diagnostic criteria. Histopathological diagnoses of moderate dysplasia, severe dysplasia, or cancer were considered neoplastic; otherwise, they were considered benign.

#### 2.3.2 Identification of regions with visible nuclei

The first step in the ND-CI algorithm (Fig. 2) is to identify regions with visible nuclei. To accomplish this, we trained, validated, and tested a convolutional neural network (CNN) that classifies each pixel within an HRME image as “visible nuclei,” “no visible nuclei,” or “background.”

Patients in the databank were randomly partitioned into training, validation, and test sets with a 50/20/30 split. To acquire a ground truth, three of the authors (E. Y., D. B., and I. V.) manually classified each pixel of each image as visible nuclei or no visible nuclei. Pixels outside the circular FOV were considered background. The ground truths were then combined into a composite ground truth; pixels deemed no visible nuclei by at least two of the three raters were set as no visible nuclei, and all other nonbackground pixels were set as visible nuclei.

The CNN architecture was based on the U-Net, an architecture popular for semantic segmentation.^23^^,^^24^ The U-Net consists of an encoder path, which captures image context information, and a decoder path, which provides localization information.^25^ To develop the CNN, multiple hyperparameters were explored, including the optimization algorithm, the learning rate and schedule, L2 regularization, batch size, and data augmentation with reflections and random crops with resizing. Various architectural parameters, including the number of layers, the channel depth of the layers, and use of batch normalization layers,^26^ were also explored. Each network was trained to minimize the cross-entropy loss, averaged across pixels in the FOVs. The contribution of no visible nuclei and visible nuclei pixels to the loss was weighted in proportion to their prevalence in the training set. Background pixels were already known and therefore did not contribute to the loss. The validation set cross-entropy loss was calculated at each epoch as the networks were trained, which continued at least until the validation loss reached a minimum. Networks were trained in Python 3.6 using the Pytorch 1.54.0 framework on a Linux desktop with two Nvidia GeForce GTX 2080 Ti GPUs.

**Fig. 3:**
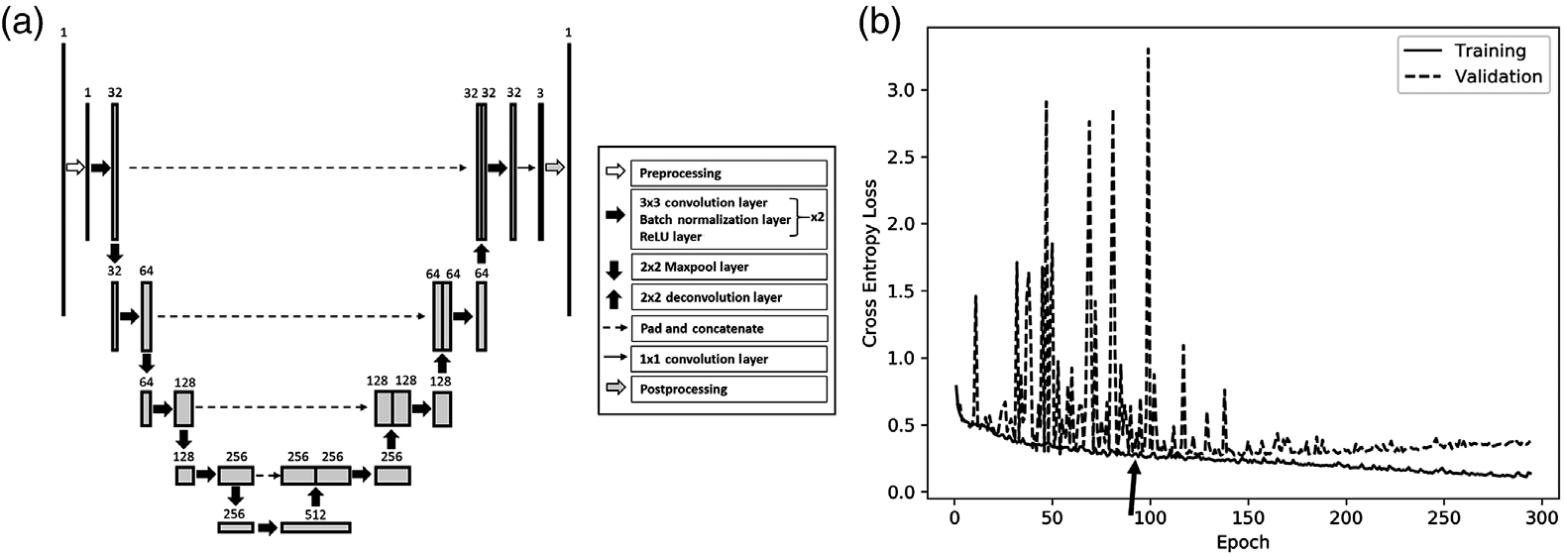
CNN architecture and training. (a) CNN architecture based on the U-Net. The arrows represent the layers of the CNN, and the rectangles represent the arrays outputted by the layers. The length of each rectangle corresponds to the height and width of the array, and the width of each rectangle represents the channel depth of the array. The channel depth is also displayed numerically above each rectangle. The HRME image (top left rectangle) is preprocessed, then fed into the encoder path (left half) and the decoder path (right half). The output (top right rectangle) is a mask of the predicted classifications for each pixel. (b) The cross-entropy loss of the selected CNN for the training and validation sets during training. Epoch 92 (arrow) was selected for prospective evaluation.

The network with the minimal validation set intersection over union (IoU; defined later in this section) was selected as the final network [Fig. 3(a)]. This network first preprocessed the input HRME image by transforming its pixel intensities from a range of [0, 1] to [−1,1] using the formula $i_{\text{new}}=2(i_{\text{old}}-0.5)$, where i was the pixel intensity (thick, unfilled right arrow). The images were then rescaled to 512×512  pixels. The preprocessed array was fed into the encoder path [Fig. 3(a), left half], which consisted of five repetitions of a six-layer group (thick, filled right arrows), with 2×2 maxpool layers (thick, filled down arrows) between each repetition. The six-layer group consisted of two repetitions of a 3×3 convolution layer with zero padding, a batch normalization layer, and a ReLU layer. The first convolution layer in each six-layer group modified the channel depth; the first convolution layer in the first six-layer group increased the channel depth from 1 to 32, and the remaining four each doubled the channel depth.

The output of the encoder path was fed into the decoder path [Fig. 3(a), right half], which consisted of a 2×2 deconvolution layer (thick, filled up arrow), followed by four repetitions of the six-layer group, with additional deconvolution layers between repetitions. The deconvolution layers halved the channel depth, and their outputs were concatenated to the encoder path array with the same channel depth (dotted arrows) before the next six-layer group. Unlike the encoder path, the first convolution in the six-layer groups halved rather than doubled the channel depth.

Next, a 1×1 convolution layer (thin, right arrow) decreased the channel depth from 32 to 3, such that each of the remaining channels represented one of the three classes (visible nuclei, no visible nuclei, and background). This three-channel array was postprocessed (thick, gray right arrow) to generate the final classification mask. Because its height and width were smaller than those of the input image due to the border pixels lost in the convolutions and initial image rescaling, bilinear interpolation was used to upsample the array to the height and width of the original input image. The softmax function was then applied to the visible nuclei and no visible nuclei channels to predict the probability that each pixel was visible nuclei or no visible nuclei. The probabilities were binarized at 0.5, and background pixels were set as background. CNN parameters were optimized with the Adam algorithm^27^ with $\alpha =1\times 10^{-4}$, $\beta_{1}=0.9$, $\beta_{2}=0.999$, and an L2 regularization penalty of $1\times 10^{-4}$. The learning rate was decayed by a factor of 0.9 every 10 epochs. The batch size was five images; data augmentation with horizontal and vertical reflections and random crops with resizing was also utilized.

The final, trained CNN was used to classify every image in the databank. Because the test set was not used to train or validate the final CNN, the test set predictions were fully prospective. To evaluate CNN performance, four metrics were calculated: the accuracy, the sensitivity, the specificity, and the IoU. The sensitivity was defined as the fraction of no visible nuclei pixels that were correctly classified, and the specificity was defined as the fraction of visible nuclei pixels that were correctly classified. The IoU is a summary metric that equals the average of the IoU of the two classes (visible nuclei and no visible nuclei). The IoU of each class is equal to the number of pixels of that class that are correctly classified, divided by the number of pixels that are, or are predicted to be, of that class. IoUs for sets of images were calculated on a pixel basis. Background pixels were not considered in performance evaluation.

#### 2.3.3 Confidence interval estimation formula

The second step in the ND-CI algorithm (Fig. 2) is to use the regions of the image with visible nuclei to calculate the CI for the “true” number of abnormal nuclei $\mathrm{per}\;{\mathrm{mm}}^{2}$, had nuclei been visible in the entire FOV. To accomplish this, we developed a formula to estimate the CI, then performed an experiment to validate the formula.

The number of abnormal nuclei $\mathrm{per}\;{\mathrm{mm}}^{2}$ within the regions with visible nuclei is p^(n^/A^), where p^ is the proportion of the nuclei within the regions that are abnormal, n^ is the total number of nuclei within the regions, and A^ is the area of the regions in ${\mathrm{mm}}^{2}$. (p^ and n^ are calculated using the methods of the ND algorithm.) Using these definitions, the density of nuclei within the regions equals (n^/A^). Similarly, the true number of abnormal nuclei $\mathrm{per}\;{\mathrm{mm}}^{2}$ equals p(n/A), where p, n, and A represent the same quantities in the full FOV. Then the uncertainty in the true number of abnormal nuclei $\mathrm{per}\;{\mathrm{mm}}^{2}$ is due to uncertainty in the proportion of nuclei that are abnormal (if p≠p^) and uncertainty in the density of nuclei (if n/A≠n^/A^). To derive the formula for the CI, we assume that n/A=n^/A^ and estimate the uncertainty in p by modeling abnormal nuclei as a binomial distribution and utilizing the Clopper–Pearson exact method for binomial proportion CIs,^28^ which calculates a CI for p at an arbitrary confidence level using n^ and p^. Then the desired CI (${\mathrm{CI}}_{\text{low}}$ to ${\mathrm{CI}}_{\text{high}}$) for the true number of abnormal nuclei $\mathrm{per}\;{\mathrm{mm}}^{2}$ is plow(n^/A^) to phigh(n^/A^).

To validate this formula, we selected 48 HRME images that had visible nuclei throughout the entire FOV and therefore had a known true number of abnormal nuclei $\mathrm{per}\;{\mathrm{mm}}^{2}$. These images were selected to have a diverse range of nuclear morphologies; the original ND algorithm classified 24 as benign, and 24 as neoplastic. 240,000 circular ROIs were randomly generated within these 48 HRME images. The 240,000 circular ROIs consisted of 200 ROIs at random locations for each of 25 possible radii, for each of the 48 images (200*25*48=240,000). The possible radii ranged from 41 pixels to the radius of the full FOV, and the center of each ROI was located such that the ROI was fully within the FOV of the image. To accomplish this, the center of each ROI was randomly located within the circle concentric to the FOV with radius $\left(R_{\mathrm{FOV}}-R_{\mathrm{ROI}}\right)$, where $R_{\mathrm{FOV}}$ and $R_{\mathrm{ROI}}$ were the radius of the FOV and the ROI, respectively. An ROI with a center outside this circle would not be fully contained within the FOV.

The nuclei within each ROI were used to estimate CIs for the true number of abnormal nuclei $\mathrm{per}\;{\mathrm{mm}}^{2}$ for the whole FOV using the formula at several confidence levels (50%, 60%, 70%, 80%, and 90%). The percentage of the CIs that included the true number of abnormal nuclei $\mathrm{per}\;{\mathrm{mm}}^{2}$, also called the coverage probability, was then calculated for each confidence level. The CI formula is valid if the coverage probability is equal to the intended confidence level.

#### 2.3.4 Testing the ND-CI algorithm

With both components developed, the full ND-CI algorithm was assessed by comparing its positive predictive value (PPV) and negative predictive value (NPV) on the test set to that of the ND algorithm, using biopsy-confirmed histopathology, where available, as the gold standard. In some cases, multiple HRME images corresponded to a single biopsy. For those biopsies, the image with the worst classification that could be made with confidence was chosen to represent the biopsy. For example, a hypothetical biopsy that corresponded to three images classified by the ND-CI algorithm as neoplastic, benign, and “cannot classify” would have an ND-CI classification of neoplastic. A biopsy corresponding to images classified by the ND-CI algorithm as benign and cannot classify would have an ND-CI classification of benign.

## 3 Results

### 3.1 Identification of Regions with Visible Nuclei

Of the 169 patients, 82 patients (from which 391 images were acquired) were assigned to the training set, 33 patients (from which 167 images were acquired) were assigned to the validation set, and 54 patients (from which 253 images were acquired) were assigned to the test set (Table 1). Seventy-nine percent of the FOVs were found to contain visible nuclei based on the composite ground truth, distributed roughly evenly among the three sets.

**Table 1:** Image databank.

	Number of patients	Number of images	Percentage of the FOVs of the images with visible nuclei
Training set	82	391	78
Validation set	33	167	81
Test set	54	253	79
Total	169	811	79

**Fig. 4:**
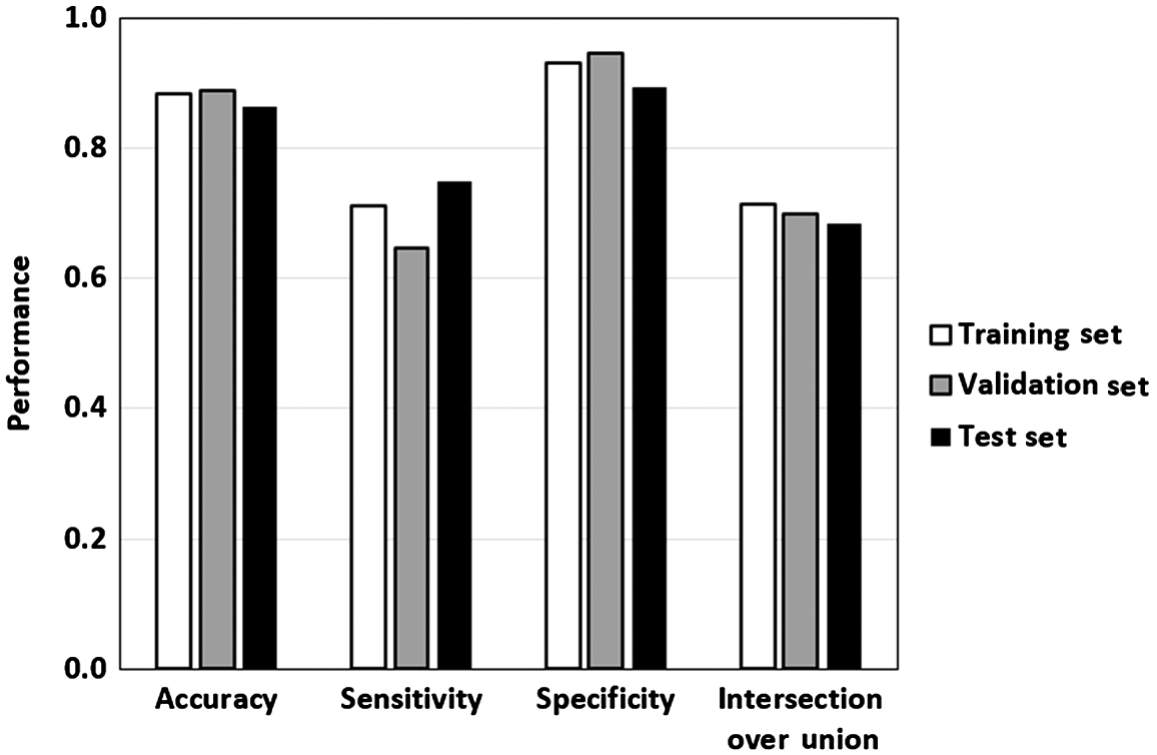
CNN performance. The accuracy, sensitivity, specificity, and IoU of the CNN in distinguishing pixels from regions with visible nuclei and regions with no visible nuclei. Regions with no visible nuclei were considered the positive class to calculate the sensitivity and specificity.

Figure 3(b) shows the cross-entropy loss of the selected CNN for the training set (solid line) and validation set (dotted line) during training. The training set loss decreased throughout training, whereas the validation set loss stabilized, and gradually began to overfit. Epoch 92 (arrow) had the highest validation IoU and was selected for prospective evaluation. The performance of the CNN was highest for the training set and slightly lower for the validation and test sets, indicating a slight overfit (Fig. 4), since the test set results were fully prospective. The accuracies for the training/validation/test sets, respectively, were 0.88/0.89/0.86, the sensitivities were 0.71/0.65/0.75, the specificities were 0.93/0.95/0.89, and the IoUs were 0.72/0.70/0.68.

### 3.2 Confidence Interval Estimation Formula

**Fig. 5:**
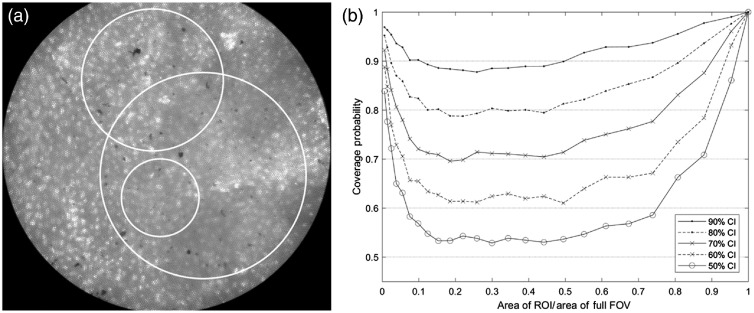
Experimental validation of CI estimation. (a) Example of an HRME image selected to validate the CI formula. The white circles are examples of the circular ROIs randomly generated within this image. (b) The coverage probability, plotted as a function of ROI area (expressed as a fraction of the area of the full FOV), for each of the intended confidence levels (horizontal dotted lines).

Figure 5(a) shows one of the 48 HRME images selected to validate the CI formula. Note that nuclei are visible in the entire FOV. Three of the 240,000 randomly positioned circular ROIs generated for this image, each with a different radius, are shown (white circles).

The coverage probabilities are plotted as a function of ROI area in Fig. 5(b) for each of the intended confidence levels, which are indicated by the horizontal dotted lines. Except for ROIs with areas <0.07 or >0.75, the coverage probabilities were close to the intended confidence levels, indicating that the CI formula is valid. ROIs with areas <0.07 or >0.75 had coverage probabilities greater than the intended confidence level, indicating that the CIs are wider than necessary.

### 3.3 Testing the ND-CI Algorithm

#### 3.3.1 Results on example images

The final two columns of Fig. 1 show the results of the ND-CI algorithm applied to the example images, all of which were in the test set and therefore fully prospective. Results of the CNN are shown in the fourth column [Fig. 1(m)–1(p)]. The main, central saturated region in the first image was correctly identified by the CNN, although the borders differed slightly [Fig. 1(m)]. The saturated and dim regions in the second image were also correctly identified, although the CNN more aggressively segmented the saturated region [Fig. 1(n)]. The CNN also accurately identified the horizontal band of nuclei in the third image [Fig. 1(o)]. Finally, the CNN appropriately determined that none of the nuclei in the fourth image [Fig. 1(p)] could be segmented.

The final column [Fig. 1(q)–1(t)] shows nuclei identified in regions with visible nuclei. The number of abnormal nuclei $\mathrm{per}\;{\mathrm{mm}}^{2}$ and 90% CIs for the images were 73 (53 to 98), 277 (233 to 325), 155 (117 to 198), and N/A (N/A to N/A). Therefore, two of the images were classified correctly, as benign [Fig. 1(q)] and neoplasia [Fig. 1(r)], whereas the other two images could not be classified with confidence [Figs. 1(s) and 1(t)].

Like the original ND algorithm, the ND-CI algorithm correctly classified the first two images. However, the ND-CI algorithm addressed issues that caused the original ND algorithm to misclassify the third and fourth images. By excluding the hyperkeratinized region of the third image, the ND-CI algorithm determined that the image could not be classified. By excluding the entire fourth image, the ND-CI determined that the image could not be classified.

#### 3.3.2 Comparison of ND algorithm and ND-CI algorithm

Eighty-two biopsies were acquired from the 54 patients in the prospective test set, of which 36 were histopathologically neoplastic and 46 were histopathologically benign. A confusion matrix summarizing the classifications of the ND and ND-CI algorithm on the test set biopsies is shown in Table 2.

**Table 2:** Results of ND algorithm and ND-CI algorithm.

	ND algorithm		ND-CI algorithm	
	Neoplastic (per pathology)	Benign (per pathology)	Neoplastic (per pathology)	Benign (per pathology)
Neoplastic (per algorithm)	27	19	17	9
Benign (per algorithm)	9	27	7	25
Cannot classify (per algorithm)	N/A	N/A	12 total including: 10 true positives 2 false negatives by ND algorithm	12 total including: 2 true negatives 10 false positives by ND algorithm

**Fig. 6:**
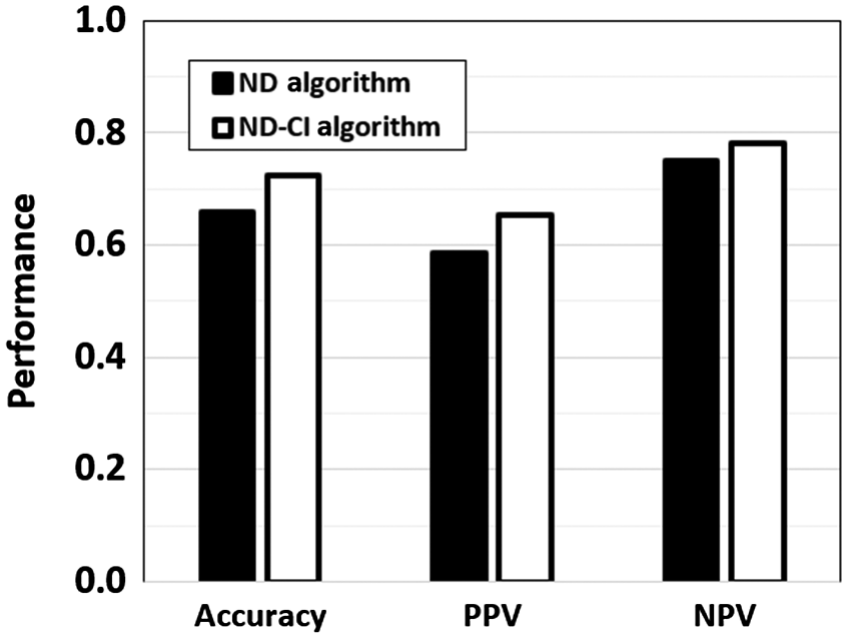
Accuracy, PPV, and NPV of the ND algorithm and ND-CI algorithm.

The ND algorithm had an accuracy of 66% (54/82), PPV of 59% (27/46), and a negative predictive value (NPV) of 75% (27/36) (Fig. 6). The ND-CI algorithm performed better, with an accuracy of 72% (42/58), PPV of 65% (17/26), and an NPV of 78% (25/32), although the differences were not statistically significant (two-tailed z-test; p=0.41, p=0.58, and p=0.76, respectively), possibly due to insufficient sample size. However, the ND-CI algorithm could not classify 24 biopsies, including one-third of the neoplasia. If the results of the ND-CI algorithm had been available in real time, it could signal the provider to acquire additional images from the same area with the goal of achieving a sufficiently small CI to predict tissue type with the desired level of confidence.

## 4 Discussion

In this paper, we developed the ND-CI algorithm to classify HRME images of the oral epithelium, including those containing regions without visible nuclei, as benign or neoplastic.

The first step of the algorithm was to train a CNN to exclude regions without visible nuclei with good performance on a prospective test set. The example images (Fig. 1) demonstrated that the CNN learned to exclude several types of regions, including saturated, dim, keratinized, and low-contrast regions. Motion blur, optical distortion, and air bubble regions were successfully excluded in other images not shown. Despite these successes, room for improvement remains. Some of the prediction masks had sharp edges and small, noisy regions, which could be addressed with postprocessing steps like removing regions below a size threshold or smoothing region borders. Performance could also be improved by utilizing strategies for training neural networks on unbalanced data, transfer learning, or more state-of-the-art network architectures.^25^^,^^29^^–^^31^

We then developed and validated a formula that used the visible nuclei to estimate the CI for the number of abnormal nuclei $\mathrm{per}\;{\mathrm{mm}}^{2}$. For ROIs with an area between 7% and 75% of the full FOV, the coverage probabilities of the CIs approximately equaled the desired confidence levels, confirming the formula’s validity. However, for small and large ROIs, the coverage probabilities were greater than the confidence levels.

These deviations can be contextualized by examining the two assumptions used to derive the formula: (1) n/A=n^/A^   (i.e., the nuclear density in the full FOV equals the nuclear density in the regions with visible nuclei) and (2) that abnormal nuclei can be accurately modeled with a binomial distribution. When the regions containing visible nuclei are small, nuclear density could differ due to chance. The second assumption that abnormal nuclei follow a binomial distribution was used to estimate a CI for p, the proportion of nuclei in the full FOV that are abnormal. The binomial distribution describes successes in samples drawn from an infinite population. For finite populations, the binomial distribution is still accurate if the sample size is small relative to the population size. For high-resolution images, the population is the nuclei in the FOV, samples are composed of visible nuclei, and a success is an abnormal nucleus. Because the number of nuclei in an image (i.e., the population) is finite, the binomial assumption loses accuracy for images with visible nuclei regions that are large relative to the area of the full FOV, resulting in excessively broad CI estimates for p. This could explain why the coverage probabilities in the validation experiment were larger than desired for large ROIs. The hypergeometric distribution, which describes successes in samples drawn from a finite population, would be a better model of abnormal nuclei. However, techniques to calculate hypergeometric proportion CIs are poorly established, complicated to implement, and computationally expensive.^32^ Alternatively, correction factors applied to the binomial distribution for finite populations meant to approximate the hypergeometric distribution^33^^,^^34^ could improve the coverage probabilities.

Images with a CI that included the decision boundary were excluded from classification by the ND-CI algorithm. This concept of classification with a reject option has been explored in other literature;^35^^–^^37^ however, the rejection criteria used by other authors differs from the CI approach used here. In those works, observations with feature(s) of interest (or a posterior probability calculated from the features) within some intermediate range of values (or probabilities) are rejected because they cannot be classified with certainty. While useful, these approaches do not account for the uncertainty in the value of the feature itself, which the CI approach is based on.

Overall, the ND-CI algorithm had better accuracy, PPV, and NPV than the original ND algorithm, when prospectively compared to histopathology, although the differences did not reach statistical significance. The improved performance was likely due to the region exclusion leading to a more accurate number of abnormal nuclei $\mathrm{per}\;{\mathrm{mm}}^{2}$, and the 90% CI estimates enabling the exclusion of images for which confident classification was not possible. If implemented in real-time, a “cannot classify” results would signal to the provider to acquire additional images with a greater number of visible nuclei. The PPV and NPV were chosen to evaluate the algorithm because they are directly relevant to this use case: they represent the performance, after an image has been deemed satisfactory. Interpreting metrics like sensitivity and specificity is less straightforward due to the “cannot classify” category.

However, the ND-CI algorithm could not classify nearly 30% of the images. In practice, the greatest effect of this was to reduce the number of false positives, although false negatives, true negatives, and true positives were also excluded. Images that could not be classified either had a number of abnormal nuclei $\mathrm{per}\;{\mathrm{mm}}^{2}$ close to the decision threshold, or a broad CI. All else equal, an image with a larger excluded region would have fewer visible nuclei, widening the CI and increasing the chance that it would fall into the “unable to classify” category. Therefore, the number of excluded images can be tuned by adjusting the CNN binarization probability (affecting size of the excluded region), or the prespecified confidence level.

Overall, the ND-CI algorithm represents an important step toward translating *in vivo* optical microscopy of oral lesions into clinical practice. The algorithm enables images with keratinized tissue and other defects to be analyzed, if possible, or informs the user that a better image is required. Although there is no guarantee that a better image can be acquired, based on our empirical experience we believe that it can be done a substantial portion of the time. If not, the algorithm is still beneficial: it informs the clinician that the imaging results should not be used to guide clinical management.

Commercially available diagnostic adjuncts for the evaluation of oral lesions include toluidine blue dye, cytology, autofluorescence imaging (AFI), and tissue reflectance imaging. In recent years, optical imaging approaches including fluorescence lifetime imaging, reflectance confocal microscopy, confocal laser microendoscopy, and optical coherence tomography have also been explored; however, these technologies are still under development and there have not been any well-designed, prospective studies to estimate their diagnostic accuracy. In 2017, an American Dental Association (ADA) panel published a meta-analysis to estimate the sensitivity and specificity of the major commercially available adjuncts on nonsuspicious and suspicious lesions.^38^ Excluding cytology, which cannot be interpreted at the point-of-care, AFI had the highest sensitivity (90%) and specificity (72%) to classify clinically suspicious lesions. However, the sensitivity and specificity of AFI were only 50% and 39%, respectively, for nonsuspicious lesions. Furthermore, the panel also found that the evidence was of low quality and volume: most studies had a high risk of bias and applicability concerns, interpretation of the adjuncts was based on subjective criteria, and for three adjunct/lesion type combinations, only a single study satisfied their inclusion criteria. Ultimately, the panel recommended against the routine use of any adjunct.^39^

Although this study was not designed to evaluate the sensitivity and specificity of HRME for clinically suspicious or nonsuspicious lesions, the performance of the HRME in this study is comparable to or exceeds that of the adjuncts included in the ADA meta-analysis. With the ND-CI algorithm, the HRME also has the added advantage of fully automated analysis and image classification.

To better understand the impact and benefits of the ND-CI algorithm, future work will include studies, in which the algorithm is used in real-time to guide image acquisition. With this additional data, techniques like ROC analysis could also be used to optimize the thresholds to tailor the performance characteristics to various clinical scenarios. For example, a high sensitivity would likely be desirable for screening.

## References

[1] Li X. D. et al., “optical coherence tomography: advanced technology for the endoscopic imaging of Barrett’s esophagus,” Endoscopy 32, 921–930 (2000). ENDCAM 10.1055/s-2000-9626 11147939

[r2] Leavesley S. J. et al., “Hyperspectral imaging fluorescence excitation scanning for colon cancer detection,” J. Biomed. Opt. 21, 104003 (2016). JBOPFO 1083-3668 10.1117/1.JBO.21.10.104003 27792808 PMC5084534

[r3] Stevenson A. D. et al., “Systematic review of diagnostic accuracy of reflectance confocal microscopy for melanoma diagnosis in patients with clinically equivocal skin lesions,” Dermatol. Pract. Concept. 3, 19–27 (2013). 10.5826/dpc.0304a05 24282659 PMC3839827

[4] Schlosser C. et al., “Fluorescence confocal endomicroscopy of the cervix: pilot study on the potential and limitations for clinical implementation,” J. Biomed. Opt. 21, 126011 (2016). JBOPFO 1083-3668 10.1117/1.JBO.21.12.126011 27999860 PMC8357321

[5] Lucchese A. et al., “The potential role of in vivo reflectance confocal microscopy for evaluating oral cavity lesions: a systematic review,” J. Oral Pathol. Med. 45, 723–729 (2016). JPMEEA 0904-2512 10.1111/jop.12454 27229884

[r6] Maher N. G. et al., “In vivo confocal microscopy for the oral cavity: current state of the field and future potential,” Oral Oncol. 54, 28–35 (2016). EJCCER 1368-8375 10.1016/j.oraloncology.2016.01.003 26786962

[r7] Muldoon T. J. et al., “Subcellular-resolution molecular imaging within living tissue by fiber microendoscopy,” Opt. Express 15, 16413 (2007). OPEXFF 1094-4087 10.1364/OE.15.016413 19550931 PMC3065245

[8] Yang E. C. et al., “Noninvasive diagnostic adjuncts for the evaluation of potentially premalignant oral epithelial lesions: current limitations and future directions,” Oral Surg. Oral Med. Oral Pathol. Oral Radiol. 125, 670–681 (2018). 10.1016/j.oooo.2018.02.020 29631985 PMC6083875

[9] Oetter N. et al., “Development and validation of a classification and scoring system for the diagnosis of oral squamous cell carcinomas through confocal laser endomicroscopy,” J. Transl. Med. 14, 159 (2016). 10.1186/s12967-016-0919-4 27255924 PMC4891821

[r10] Olsovsky C. et al., “Handheld tunable focus confocal microscope utilizing a double-clad fiber coupler for in vivo imaging of oral epithelium,” J. Biomed. Opt. 22, 056008 (2017). JBOPFO 1083-3668 10.1117/1.JBO.22.5.056008 PMC5444308 28541447

[r11] Malik B. H. et al., “A novel multimodal optical imaging system for early detection of oral cancer,” Oral Surg. Oral Med. Oral Pathol. Oral Radiol. 121, 290–300.e2 (2016). 10.1016/j.oooo.2015.10.020 26725720 PMC4752880

[r12] Pierce M. C. et al., “Accuracy of in vivo multimodal optical imaging for detection of oral neoplasia,” Cancer Prev. Res. 5, 801–809 (2012). 10.1158/1940-6207.CAPR-11-0555 PMC3560936 22551901

[r13] Quang T. et al., “Prospective evaluation of multimodal optical imaging with automated image analysis to detect oral neoplasia in vivo,” Cancer Prev. Res. (Phila). 10, 563–570 (2017). 10.1158/1940-6207.CAPR-17-0054 28765195 PMC5626620

[r14] Yang E. C. et al., “In vivo multimodal optical imaging: improved detection of oral dysplasia in low-risk oral mucosal lesions,” Cancer Prev. Res. (Phila). 1, 465–476 (2018). 10.1158/1940-6207.CAPR-18-0032 PMC6082417 29903741

[r15] Yang E. C. et al., “Prospective evaluation of oral premalignant lesions using a multimodal imaging system: a pilot study,” Head Neck 42, 171–179 (2020). 10.1002/hed.25978 31621979 PMC7003735

[16] Nathan C.-A. O. et al., “Confocal laser endomicroscopy in the detection of head and neck precancerous lesions,” Otolaryngol. Head. Neck Surg. 151, 73–80 (2014). OHNSDL 0194-5998 10.1177/0194599814528660 24699456

[17] Dittberner A. et al., “Automated analysis of confocal laser endomicroscopy images to detect head and neck cancer,” Head Neck 38, E1419–E1426 (2016). 10.1002/hed.24253 26560348

[r18] Harris M. A. et al., “Pulse coupled neural network segmentation algorithm for reflectance confocal images of epithelial tissue,” PLoS One 10, e0122368 (2015). POLNCL 1932-6203 10.1371/journal.pone.0122368 25816131 PMC4376773

[19] Quang T. et al., “A tablet-interfaced high-resolution microendoscope with automated image interpretation for real-time evaluation of esophageal squamous cell neoplasia,” Gastrointest. Endosc. 84, 834–841 (2016). 10.1016/j.gie.2016.03.1472 27036635 PMC5045314

[20] Yang E. C. et al., “Development of an integrated multimodal optical imaging system with real-time image analysis for evaluation of oral premalignant lesions,” J. Biomed. Opt. 24, 1 (2019). JBOPFO 1083-3668 10.1117/1.JBO.24.2.025003 PMC6383051 30793567

[21] Lee J.-J. et al., “Factors associated with underdiagnosis from incisional biopsy of oral leukoplakic lesions,” Oral Surgery, Oral Med. Oral Pathol. Oral Radiol. Endodontol. 104, 217–225 (2007). 10.1016/j.tripleo.2007.02.012 17560138

[22] Gale N. Poljak M. Zidar N., “Update from the 4th Edition of the World Health Organization classification of head and neck tumours: what is new in the 2017 WHO Blue Book for tumours of the hypopharynx, larynx, trachea and parapharyngeal space,” Head Neck Pathol. 11, 23–32 (2017). 10.1007/s12105-017-0788-z 28247231 PMC5340729

[23] Ronneberger O. Fischer P. Brox T., U-Net: Convolutional Networks for Biomedical Image Segmentation, pp. 234–241, Springer, Cham (2015).

[24] Falk T. et al., “U-Net: deep learning for cell counting, detection, and morphometry,” Nat. Methods 16, 67–70 (2019).1548-7091 10.1038/s41592-018-0261-2 30559429

[25] Garcia-Garcia A. et al., “A review on deep learning techniques applied to semantic segmentation,” arXiv preprint arXiv:1704.06857 (2017).

[26] Ioffe S. Szegedy C., “Batch normalization: accelerating deep network training by reducing internal covariate shift,” arXiv preprint arXiv:1502.03167 (2015).

[27] Kingma D. P. Ba J., “Adam: a method for stochastic optimization,” arXiv preprint arXiv:1412.6980 (2014).

[28] Clopper C. J. Pearson E. S., “The use of confidence or fiducial limits illustrated in the case of the binomial,” Biometrika 26, 404 (1934). BIOKAX 0006-3444 10.1093/biomet/26.4.404

[29] Jégou S. et al., “The one hundred layers tiramisu: fully convolutional DenseNets for semantic segmentation,” in Proc. IEEE Conf. Comput. Vision and Pattern Recognit. Workshops (2017).

[r30] Lin G. et al., “RefineNet: multi-path refinement networks for high-resolution semantic segmentation,” in Proc. IEEE Conf. Comput. Vision and Pattern Recognit. (2017).

[31] Paszke A. et al., “ENet: a deep neural network architecture for real-time semantic segmentation,” arXiv preprint arXiv:1606.02147 (2016).

[32] Wang W., “Exact optimal confidence intervals for hypergeometric parameters,” J. Am. Stat. Assoc. 110, 1491–1499 (2015). 10.1080/01621459.2014.966191

[33] Sandiford P. J., “A new binomial approximation for use in sampling from finite populations,” J. Am. Stat. Assoc. 55, 718–722 (1960). 10.1080/01621459.1960.10483371

[34] Burstein H., “Finite population correction for binomial confidence limits,” J. Am. Stat. Assoc. 70, 67–69 (1975). 10.1080/01621459.1975.10480263

[35] Bartlett P. L. Wegkamp M. H., “Classification with a reject option using a hinge loss,” J. Mach. Learn. Res. 9, 1823–1840 (2008).

[r36] Pillai I. Fumera G. Roli F., “Multi-label classification with a reject option,” Pattern Recognit. 46, 2256–2266 (2013). 10.1016/j.patcog.2013.01.035

[37] Herbei R. Wegkamp M. H., “Classification with reject option,” Can. J. Stat. 34, 709–721 (2006). 10.1002/cjs.5550340410

[38] Lingen M. W. et al., “Adjuncts for the evaluation of potentially malignant disorders in the oral cavity: diagnostic test accuracy systematic review and meta-analysis—a report of the American Dental Association,” J. Am. Dental Assoc. 148, 797–813.e52 (2017). 10.1016/j.adaj.2017.08.045 PMC7366378 29080605

[39] Lingen M. W. et al., “Evidence-based clinical practice guideline for the evaluation of potentially malignant disorders in the oral cavity: a report of the American Dental Association,” J. Am. Dent. Assoc. 148, 712–727.e10 (2017). 10.1016/j.adaj.2017.07.032 28958308

